# Inactivation of Severe Acute Respiratory Coronavirus Virus 2 (SARS-CoV-2) and Diverse RNA and DNA Viruses on Three-Dimensionally Printed Surgical Mask Materials

**DOI:** 10.1017/ice.2020.417

**Published:** 2020-08-12

**Authors:** Jennifer L. Welch, Jinhua Xiang, Samantha R. Mackin, Stanley Perlman, Peter Thorne, Patrick O’Shaughnessy, Brian Strzelecki, Patrick Aubin, Monica Ortiz-Hernandez, Jack T. Stapleton

**Affiliations:** 1 Medical Service, Iowa City Veterans’ Affairs Medical Center, Iowa City, Iowa; 2 Department of Internal Medicine, Carver College of Medicine University of Iowa, Iowa City, Iowa; 3 Department of Microbiology and Immunology, Carver College of Medicine, University of Iowa, Iowa City, Iowa; 4 Department of Occupational and Environmental Health, College of Public Health, University of Iowa, Iowa City, Iowa; 5 VA Puget Sound Health Care System, Seattle, Washington; 6 Center for Limb Loss and MoBility (CLiMB), VA Puget Sound Health Care System, Seattle, Washington; 7 Department of Mechanical Engineering, University of Washington, Seattle, Washington

## Abstract

**Background:**

Personal protective equipment (PPE) is a critical need during the coronavirus disease 2019 (COVID-19) pandemic. Alternative sources of surgical masks, including 3-dimensionally (3D) printed approaches that may be reused, are urgently needed to prevent PPE shortages. Few data exist identifying decontamination strategies to inactivate viral pathogens and retain 3D-printing material integrity.

**Objective:**

To test viral disinfection methods on 3D-printing materials.

**Methods:**

The viricidal activity of common disinfectants (10% bleach, quaternary ammonium sanitizer, 3% hydrogen peroxide, or 70% isopropanol and exposure to heat (50°C, and 70°C) were tested on four 3D-printed materials used in the healthcare setting, including a surgical mask design developed by the Veterans’ Health Administration. Inactivation was assessed for several clinically relevant RNA and DNA pathogenic viruses, including severe acute respiratory coronavirus virus 2 (SARS-CoV-2) and human immunodeficiency virus 1 (HIV-1).

**Results:**

SARS-CoV-2 and all viruses tested were completely inactivated by a single application of bleach, ammonium quaternary compounds, or hydrogen peroxide. Similarly, exposure to dry heat (70°C) for 30 minutes completely inactivated all viruses tested. In contrast, 70% isopropanol reduced viral titers significantly less well following a single application. Inactivation did not interfere with material integrity of the 3D-printed materials.

**Conclusions:**

Several standard decontamination approaches effectively disinfected 3D-printed materials. These approaches were effective in the inactivation SARS-CoV-2, its surrogates, and other clinically relevant viral pathogens. The decontamination of 3D-printed surgical mask materials may be useful during crisis situations in which surgical mask supplies are limited.

Severe acute respiratory syndrome coronavirus 2 (SARS CoV-2) recently emerged as a highly transmissible human pathogen that rapidly escalated into a global pandemic.^1^ SARS CoV-2 is the causative agent of coronavirus disease 2019 (COVID-19), causing significant respiratory distress and mortality accounting for >18.1 million confirmed cases and ~691,000 deaths worldwide as of August 4, 2020.^2^ Six pathogenic coronaviruses are known to infect humans. Of these, SARS CoV-1, Middle East respiratory syndrome coronavirus (MERS-CoV), and now SARS CoV-2 are considered highly pathogenic.^3,4^ Human-to-human transmission of SARS CoV-2 occurs at an elevated rate compared to SARS CoV, which shares considerable sequence homology (79%).^4–6^

Until there is an effective vaccine and/or therapeutic approach to treat COVID-19, SARS-CoV-2 control strategies are focused on transmission prevention, including social distancing, hand washing, and use of personal protective equipment (PPE).^7^ The increased demand for and shortages of PPE in healthcare and other essential workplace settings has created a need to address decontamination strategies and reuse of PPE. The Veterans’ Health Administration (VHA) recently began developing a supplemental surgical mask.^8^ The use of 3-dimensional (3D) printing technology within the healthcare industry is not new; it is currently used in applications such as drug delivery systems,^9,10^ surgery,^11,12^ personalized medicine,^13–15^ and biomedical engineering.^16,17^ In response to current needs during the COVID-19 pandemic, 3D printed technology has expanded to include the production of ventilators and other respiratory support equipment,^18,19^ nasopharyngeal swabs,^20^ face shields,^21^ and face masks.^22,23^

In 2006, the US Department of Health and Human Services (DHHS) asked the Institute of Medicine (IOM) to convene a committee to conduct an evaluation of measures that would permit the reuse of disposable N95 respirators and reusable face masks in healthcare settings. The IOM committee followed criteria to prevent transmission of the 2003 SARS-CoV. The IOM committee found no validated method of decontamination that met criteria for decontamination of N95 respirators or surgical masks.^24^

Although reuse of PPE masks was considered before the SARS-CoV-2 pandemic, literature on potential decontamination strategies are limited and guidelines are often institution- or manufacturer specific.^25,26^ In this study, we assessed the ability of materials used in the 3D printing of surgical masks to be decontaminated for human viral pathogens including SARS-CoV-2 using common disinfection methods. Data showing that human coronaviruses may survive on various surfaces for up to 9 days makes identification of decontamination strategies against pathogenic viruses increasing important not only during the SARS-CoV-2 pandemic but also for future viral transmission prevention practices.^27^ Our goal was to identify practical decontamination procedures that are easily adapted across healthcare and other workplace settings. We assessed the VHA 3D-printed mask material and 3 additional 3D-printing materials for virus inactivation in these studies.

## Materials and methods

Healthy volunteers were invited to participate in the study. Following written informed consent, anticoagulated blood was obtained using heparin collection tubes. This study was approved by the University of Iowa Institutional Review Board.

We used numerous viruses in these studies. Enterovirus 68 (EV68), vaccinia virus, influenza A H1N1 and human coronavirus (HCoV-229E) were all supplied by the American Type Culture Collection (ATCC). We also tested yellow fever virus (YFV, provided by Sanofi), HIV-1 (provided by the NIH AIDS Reagent Program), mumps virus (provided by Merck), adenovirus (provided by University of Iowa Viral Vector Core), Zika virus (kindly provided by DrWendy Maury, University of Iowa), SARS CoV-2 (Seattle Washington strain MN985325), murine hepatitis virus (MHV A59 strain, kindly provided by Dr Stanley Perlman, University of Iowa), and Dengue viruses types 1–4 (DENV, kindly provided by Sarah George, St Louis University). Virus titers were determined in appropriate cell lines by median tissue culture infectious dose (TCID_50_) or p24 enzyme-linked immunosorbent assay (ELISA) for HIV-1.^28,29^ All SARS-CoV-2 work was performed at the University of Iowa Biosafety Level (BSL) 3 core facility, and all other virus studies were conducted at the Iowa City Veterans’ Affairs Infectious Diseases Research Laboratory under BSL2 conditions.

Vero and MDCK cells were purchased from the ATCC, and HEK293 and MT-2 cells were obtained from the NIH AIDS Reagent Program. MRC-5 was purchased from Sigma-Aldrich (St Louis, MO), and VeroE6 and 17Cl-1 were provided by Dr Stanley Perlman. Cells were maintained in media as previously described.^28,30-32^

The VHA supplemental surgical face mask^8^ being developed is 3D printed using Multi-Jet Fusion (MJF) technology and a powder-based polyamide-12 (PA12) material (HP 3D HR CB PA 12 - Hewlett-Packard, Palo Alto, CA). The surgical face mask incorporates a removable filter (not tested in this study). The 3D-material is a biocompatible thermoplastic used in medical applications^33^ and is believed to be resistant to disinfectants. Thus, it may be reused many times following standard approaches to disinfection. The surgical face mask design has undergone review in a clinical setting and was found appropriate when fabricated with the printer type and materials specified.^23^

Virus titers were determined by determining the TCID_50_ in the cognate cell line as previously described^28,34,35^ or by p24 ELISA (R&D Systems) for HIV-1.^29^

### Inactivation studies

Disks were printed in Seattle and shipped to Iowa for testing. Each virus (100 µL) was added to 2 cm diameter × 1.5-mm-thick 3-D printed circular disks (PA12 material unless otherwise noted). Disks were allowed to dry in a laminar flow safety cabinet at room temperature for 2 hours. Disks were inactivated either by thermal or chemical treatment (as described in the Results section). Viruses were recovered by placing the disks into a 12-well culture plate, adding 200 µL media to each well, pipetting 6 times, then removing the media and storing at −80°C until determining the viral titer (within 1 week in all cases). Virus infectivity following each inactivation method was compared to control disks treated only with phosphate-buffered saline (PBS), and the reduction in infectivity (log_10_ or p24) was calculated. Control and postinactivation virus titers were performed in a minimum of 3 biological replicate experiments.

### Statistical analysis

Statistical analyses were performed using GraphPad software V8.2 (GraphPad Prism). We used 2-tailed Student *t* tests to compare results between treatment and control virus titers from triplicate experiments. *P* < .05 was considered statistically significant.

## Results

To determine the effectiveness of inactivation of viruses applied to 3D printing material, we examined a diverse group of human viral pathogens that have different virion structures (envelope vs nonenveloped), different genome compositions (DNA vs RNA), and genome structures (single strand vs double strand).^36^ In light of the COVID-19 epidemic, we included 1 human low pathogenicity coronavirus (229E), 1 nonhuman coronavirus (MHV), and SARS-CoV-2 in most inactivation experiments. We also included a variety of healthcare-relevant human pathogens (Table 1).

**Table 1.** Viruses Used in Inactivation Studies

Virus	Strain	Genome	Envelope	Size (nm)	Cell Type
SARS CoV-2	Seattle	RNA SS +	Y	65–125	VeroE6
HCoV	229E	RNA SS +	Y	80–150	MRC-5
MHV	A59	RNA SS +	Y	80–160	17Cl-1
Mumps	Jeryl Lynn strain	RNA SS −	Y	100–600	Vero
YFV	17D strain	RNA SS +	Y	25–65	Vero
Vaccinia	Walter Reed	DNA DS	Y	200–300	Vero
Adenovirus	Type 5	DNA DS	N	70–100	HEK293
EV68	US/IL/14-18952	RNA SS +	N	25–30	MRC-5
Zika	PR	RNA SS +	Y	40–50	Vero
Dengue	Serotypes 1-4	RNA SS +	Y	40–60	Vero
Influenza A H1N1	A/Virginia/ATCC2/2009	RNA SS −	Y	80–120	MDCK
HIV-1	NL4-3 gp41 (36G) N42S	RNA SS +	Y	110–145	MT-2

Note. SS, single strand; DS, double strand; +, positive polarity; −, negative polarity; Y, enveloped virus; N, nonenveloped virus; HCoV human coronavirus; MHV, mouse hepatitis virus; YFV, yellow fever virus; EV68, enterovirus 68; HIV-1, human immunodeficiency virus type 1.

### Chemical inactivation

Chemical inactivation studies were performed by treating the virus-coated disks with a single application (by wipe) of bleach (10%; 0.6% hypochlorite), isopropyl alcohol (70% IPA), a commercial quaternary ammonium compound (Sani-Cloth germicidal disposable wipe AF3; n-Alkyl [68% C_12_, 32% C_14_] dimethyl ethylbenzyl ammonium chlorides – 0.14%; n-Alkyl [60% C_14_, 30% C_12_, 5% C_18_] dimethyl benzyl ammonium chlorides – 0.14%), control wipe (PBS), or a no-wipe control as indicated. After a single application, disks were allowed to dry (<5 minutes in all cases) and virus recovery was measured.

**Fig. 1.**
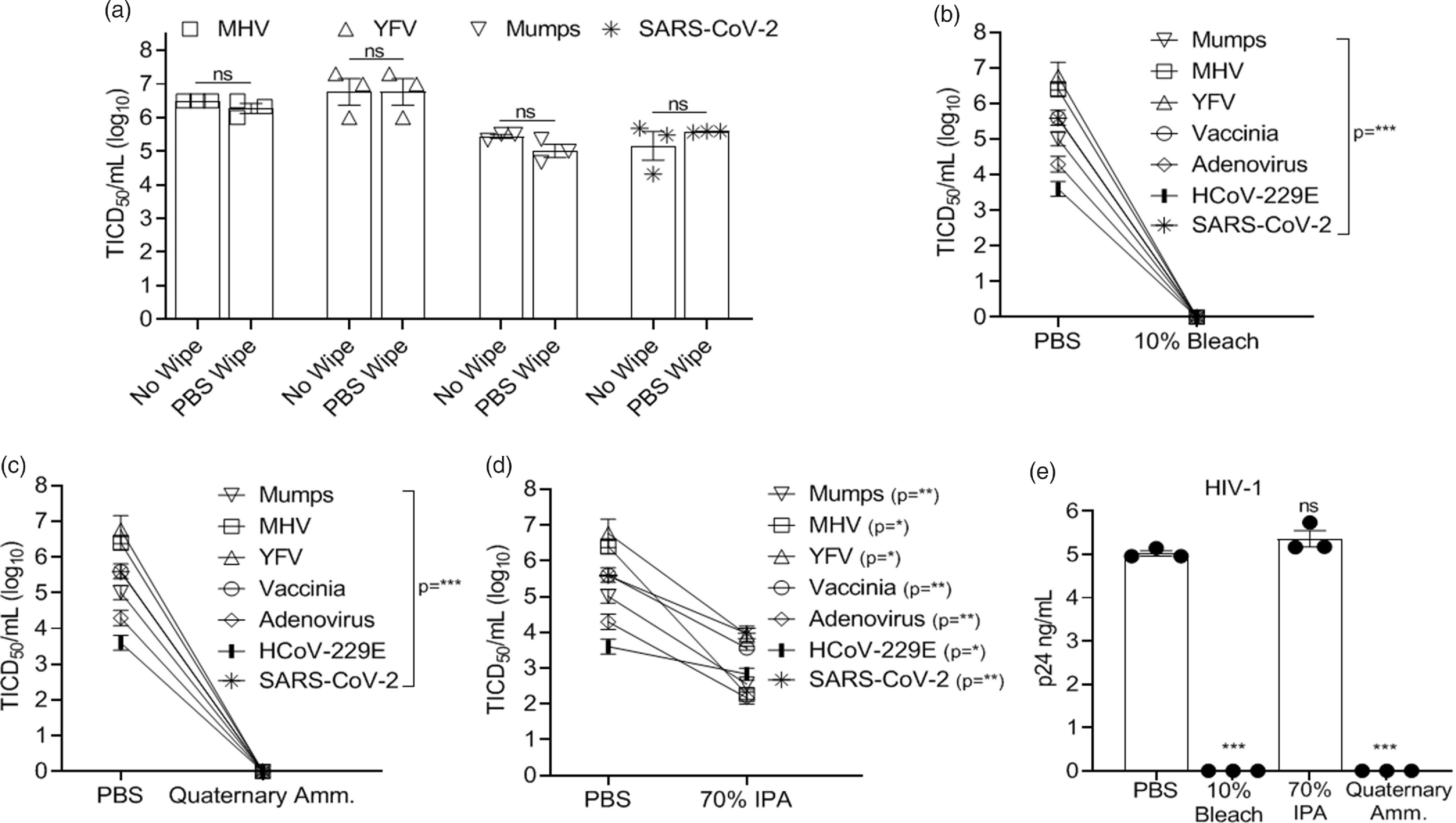
Virus inactivation (log_10_) on 3D-printed materials by method and type of chemical disinfection. Recovery of virus from 3D-printed mask material after exposure to a single-wipe application of (A) phosphate-buffered saline (PBS), (B) 10% bleach, (C) ammonium quaternary compounds, and (D) 70% IPA. (E) Recovery of HIV-1. Virus titer was completed as described in the methods in the cell typed indicated in Table 1. Significance was determined using the Student *t* test. *P < .05; **P < .01; ***P < .001; ****P < .0001; ns, not significant. Error bars represent standard error of the mean (SEM) of 3 independent experiments. Note. TCID50, median tissue culture infectious dose, MHV, mouse hepatitis virus, YFV, yellow fever virus, IPA, isopropyl alcohol. HIV-1, human immunodeficiency virus type 1.

Nearly complete recovery of virus from 3D-printed surgical mask materials was observed, and wiping the disk with PBS did not significantly reduce virus titers compared to the no-wipe controls (>99% of input virus recovered) (Fig. 1A). All viruses tested were exquisitely sensitive to bleach and quaternary ammonium compounds (Fig. 1B–C, 1E), and no infectivity remained following a single wipe of these disinfectants across the 3D mask material. In contrast, 70% IPA did not eliminate viral infectivity although there was >95% (≥1.3 log) inactivation of viruses applied with the exception of HIV-1, for which IPA was ineffective (Fig. 1D and 1E). The log_10_ reduction in infectivity is shown in Table 2.

**Table 2.** Virus Infectivity Reduction (log_10_) by Treatment^a^

Virus	Treatment		
	10% Bleach	70% IPA	Ammonium Quaternary
MHV	>6.0	2.7	>6.0
YFV	>6.5	1.7	>6.5
Mumps	>5.0	1.9	>5.0
Vaccinia	>5.5	1.6	>5.5
Adenovirus	>4.0	2.0	>4.0
229E	>3.5	1.3	>3.5
SARS-CoV-2	>5.5	1.4	>5.5

Note. IPA, isopropyl alcohol; MHV, murine hepatitis virus; YFV, yellow fever virus.

a Values represent the log_10_ reduction in infectivity following a single wipe application of the indicated treatment. Log reduction between viruses varied due to differences in virus inocula.

**Fig. 2.**
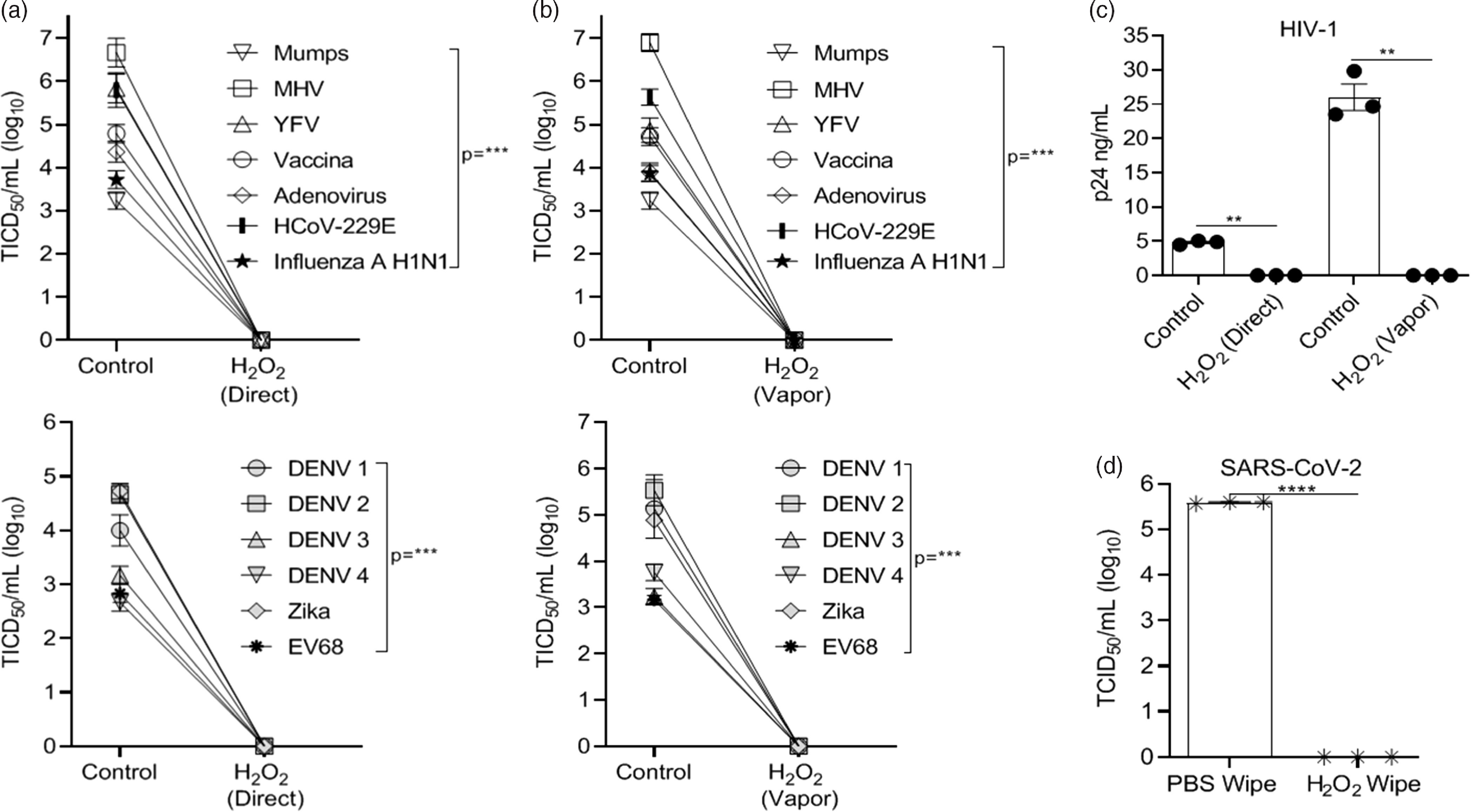
Hydrogen peroxide (H_2_O_2)_ inactivation on 3D-print material by application method versus controls. Virus recovery from 3D printed mask material after exposure to ionized H_2_O_2_ (3%) in (A) direct contact or (B) vaporized application format. (C) Recovery of HIV-1. Virus applied to 3D material without exposure to H_2_O_2_ acted as control. (D) Recovery of SARS-CoV-2 virus from 3D-printed mask material after exposure to a single-wipe application of phosphate-buffered saline (PBS) or H_2_O_2_ (3%). Virus titer was completed as described in the Methods section in the cell type indicated in Table 1. Significance was determined using the Student *t* test. *P < .05; **P< .01; ***P < .001; ****P < .0001; ns, not significant. Error bars represent standard error of the mean (SEM) of triplicate experiments. Note. DENV, dengue virus.

Vaporized H_2_O_2_ treatment of virus-coated disks utilized ionized H_2_O_2_ (~3% after application) for 15 minutes of vapor contact time and 15-minute air exchange using the SteraMist Binary Ionization Technology (BIT) at the University of Iowa Hospitals and Clinics Central Sterilizing Services (UIHC) in a 1.90 M L × 1.90 M H × 0.99 M W chamber. Alternatively, virus-coated disks were treated with ionized H_2_O_2_ (~3% after application) in a direct contact format at a 61-cm (24-inch) distance with a minimum contact time of 4 seconds. Ionized H_2_O_2_ delivery was generated by passing a low-concentration source liquid (7.8% H_2_O_2_) through a 17,000V cold plasma arc.^37^ Both vaporized and direct contact H_2_O_2_ completely inactivated all viruses (Fig. 2A–C). Notably, we were not able to test inactivation by ionized H_2_O_2_ with SARS-CoV-2 in the BSL3 facility. To assess the effect of H_2_O_2_ on SARS CoV-2, we applied 3% H_2_O_2_ to the disks with a single wipe as was done with bleach, IPA, and quaternary ammonium compounds. SARS-CoV-2 was completely inactivated by H_2_O_2_ (Fig. 2D) using this approach.

### Thermal inactivation

**Fig. 3.**
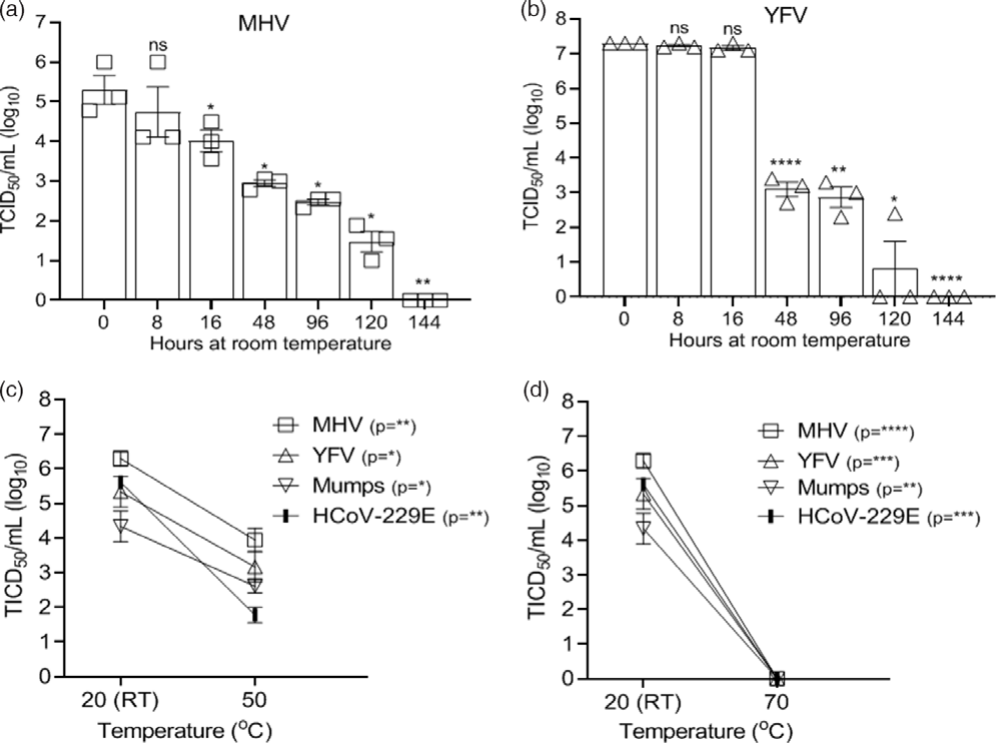
Effectiveness of virus thermal inactivation. Recovery of virus over time from 3D-printed mask material incubated at room temperature (RT, 20°C) for (A) MHV and (B) YFV. Recovery of virus from 3D material after thermal inactivation for 30 minutes at (C) 50°C or (D) 70°C. Virus incubated at 20°C (RT) acted as the control. Virus titer was completed as described in the Methods section in the cell type indicated in Table 1. Significance was determined using the Student *t* test. *P < .05; **P< .01; ***P < .001; ****P < .0001; ns, not significant. Error bars represent standard error of the mean (SEM) of triplicate experiments.

The effect of thermal inactivation on select viruses was examined by incubating the disks at room temperature and measuring virus recovery over time for the murine coronavirus serving as a SARS CoV-2 surrogate (MHV) and other virus controls (YFV). As seen in Figure 3A and B, MHV and YFV both required 6 days before infectivity was completely inactivated in these high-titer virus preparations. We tested select viruses (HCoV-229E, MHV, YFV, and mumps) for inactivation at 50°C and 70°C incubation for 30 minutes. Complete inactivation occurred for all viruses incubated at 70°C. HCoV-229E, MHV, YFV, and mumps viruses incubated at 50°C were not completely inactivated, although infectivity was reduced by >97% (1.6 log) (Fig. 3C–D). We did not have the equipment to allow testing of SARS-CoV-2 in the BSL3 at these temperatures.

### Effects of blood and repetitive disinfection on chemical inactivation

**Fig. 4.**
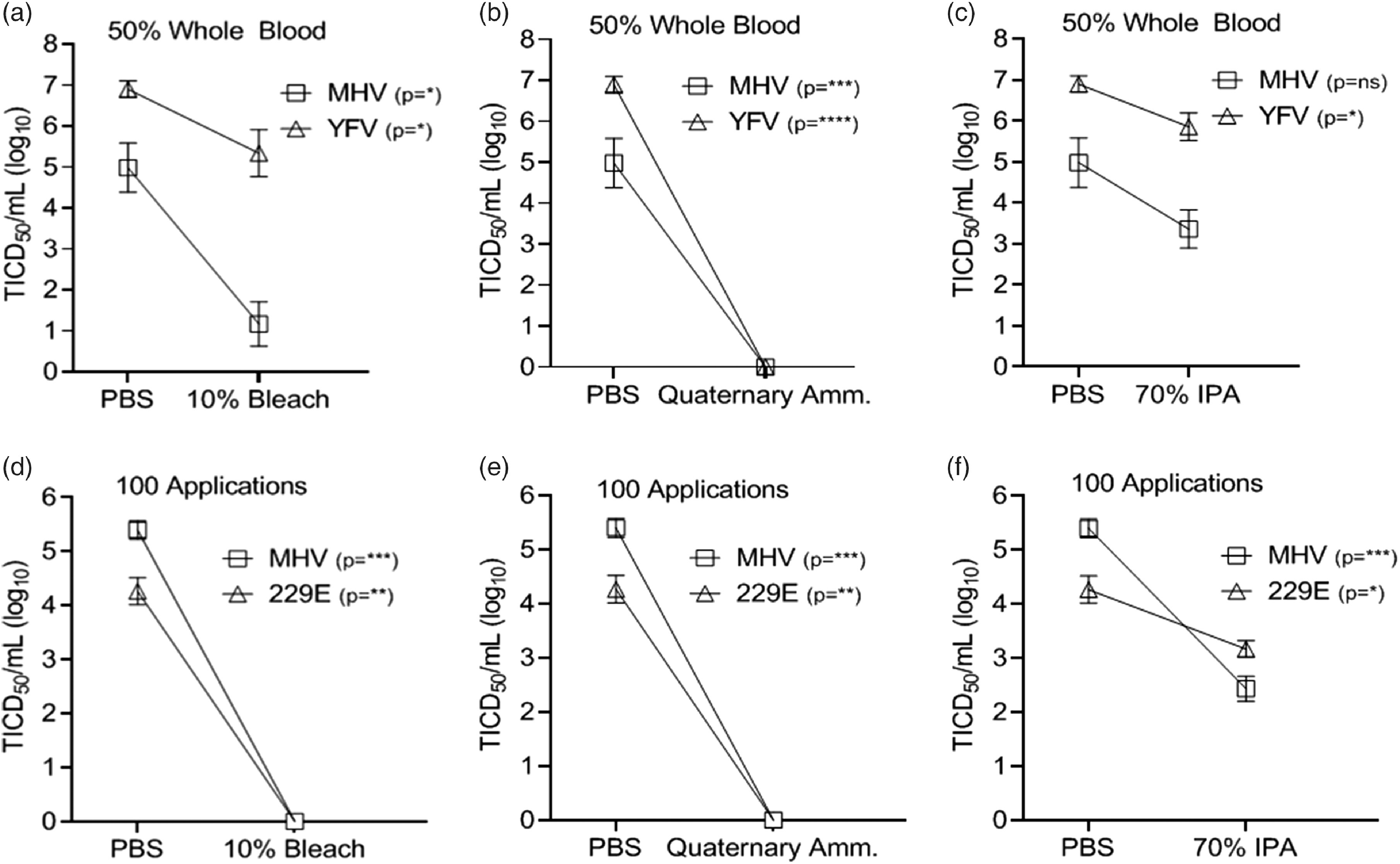
Effectiveness of chemical inactivation of viruses (log_10_) in the presence of blood and after repetitive disinfection of material. Virus recovery from 3D printed mask material after virus addition to whole blood (50% final concentration) and exposure to a single-wipe application of (A) 10% bleach, (B) ammonium quaternary, and (C) 70% IPA. Wipe application of PBS acted as control. Virus recovery from 3D material after the material was exposed to disinfectant 100 times prior to application of virus and a single-wipe application of (D) 10% bleach, (E) quaternary ammonium, and (F) 70% IPA. Virus titer was completed as described in the Methods section in the cell type indicated in Table 1. Significance was determined using the Student *t* test. *P < .05; **P< .01; ***P < .001; ****P < .0001; ns, not significant. Error bars represent standard error of the mean (SEM) of triplicate experiments.

Previous studies found that the presence of human blood may interfere with the viricidal activity of disinfectants.^38^ The effects of blood on chemical inactivation were studied by adding virus to whole blood (50% final concentration) and testing inactivation as described above. Although disinfectant sensitivity varied between viruses in the presence of blood, viruses remained highly sensitive to bleach and quaternary ammonium (Fig. 4A–B) with infectivity reduced by >93% (>1.2 log). IPA (70%) did not completely inactivate the virus preparations but did reduce infectivity by at least 92% (1.1 log) (Fig. 4C). Notably, for the MHV–blood mixture, one operator had complete inactivation, while a different operator detected a 99.9% (3 log_10_) reduction in inactivation with bleach application, highlighting the importance of maximizing inactivation.

An important concern for the reuse of PPE is the PA12 material’s stability and resistance to disinfectants. To address the effect of repeat exposure to disinfectants on efficacy of viral inactivation, the PA12 material was exposed to 100 applications of disinfectant before virus was applied and virus inactivation assessed using a single-wipe application as described above. Following repetitive inactivation, there was no grossly apparent loss of integrity, and virus inactivation was reproducible (Fig. 4D–F).

### Alternative 3D printing materials

**Fig. 5.**
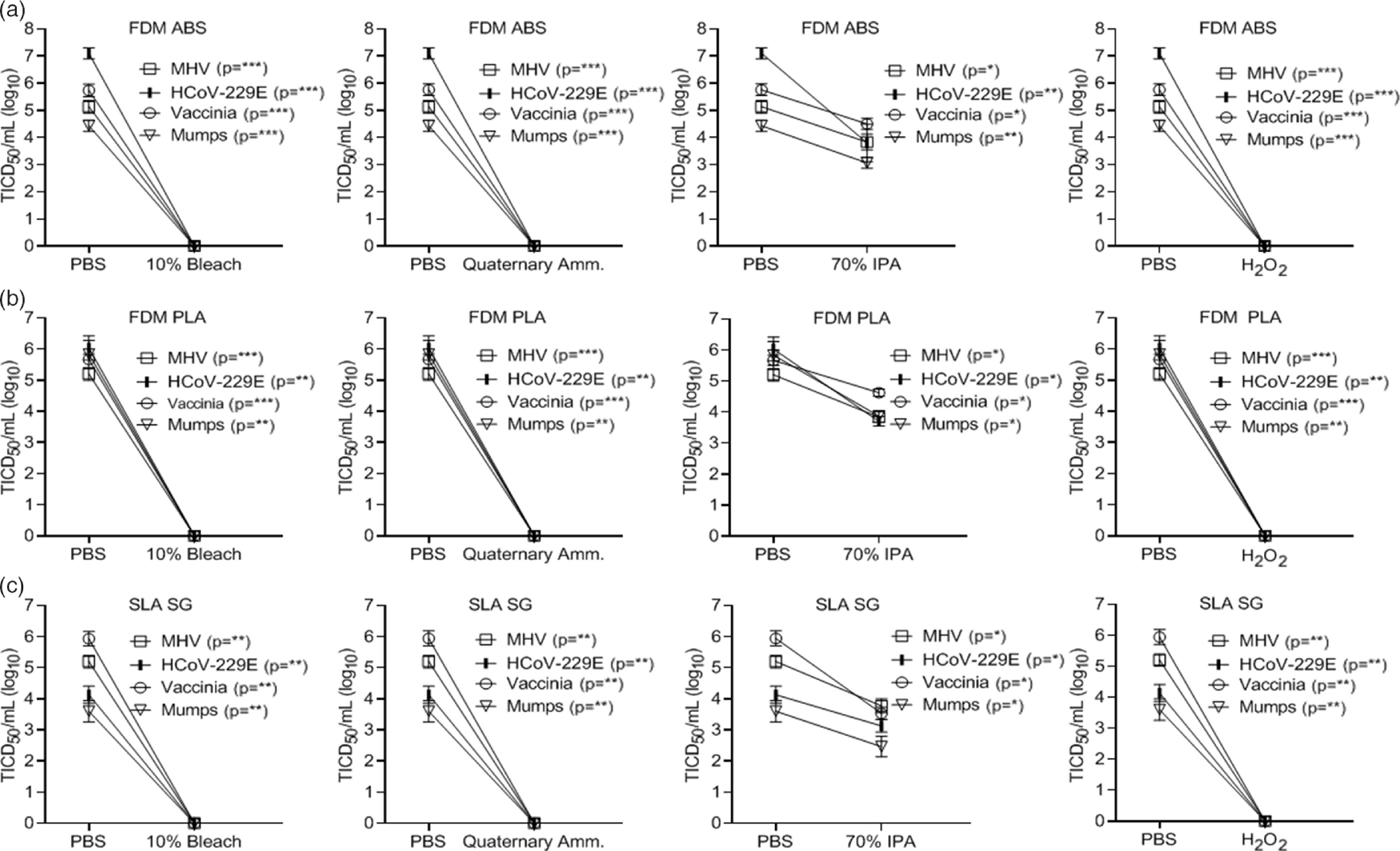
Chemical inactivation of viruses (log_10_) on alternative 3D-printed mask materials by type of disinfectant versus controls. Virus recovery from alternative 3D-printed mask material (A) FDM acrylonitrile butadiene styrene (FDM ABS), (B) FDM polylactic acid (PLA), and (C) stereolithography acrylic-surgical guide (SLA SG) after exposure to a single-wipe application of 10% bleach, ammonium quaternary, 70% IPA, and 3% H_2_O_2._ Wipe application of PBS acted as the control. Virus titer was completed as described in the Methods section in the cell type indicated in Table 1. Significance was determined using the Student *t* test. *P < .05; **P< .01; ***P < .001; ****P < .0001; ns, not significant. Error bars represent standard error of the mean (SEM) of triplicate experiments.

In addition to the PA12 material, we tested the inactivation of select viruses on 3 materials commonly used in 3D printing applications. These included a fused deposition modeling (FDM acrylonitrile butadiene styrene (ABS) material (ABS M-30 from Stratasys, Rehovot, Israel), an FDM polylactic acid (PLA) material (PLA from Stratasys, Rehovot, Israel), and a stereolithography acrylic (SLA) material (Surgical Guide from Formlabs, Somerville, MA). These additional materials were selected because the FDM and SLA 3D-printing technology platforms are very frequently utilized, and ABS and PLA are the 2 most common materials for FDM. Further, the surgical guide (SG) material is a very popular SLA material for additional medical applications. Although these 3 materials and PA12 represent the most commonly used materials, other 3D printing materials have been utilized in the healthcare setting. Application of select viruses to these materials found that SLA was more impermeable, requiring longer time intervals for virus drying (>4 hours), while PLA was permeable to the applied virus preparations. In contrast, ABS was similar to the PA12 material studied above. Virus inactivation by chemical disinfection was identical to that observed using the PA12 material (Fig. 5A–C). However, the permeability of PLA to the virus preparation would preclude use in PPE manufacture.

## Discussion

The shortage of PPE during the COVID-19 pandemic created a critical need for the development of alternative, reusable equipment. However, decontamination guidelines for reusable equipment have not reached consensus and are often limited by incomplete testing against surrogate pathogens. To address the shortage of respiratory PPE, the VHA developed a 3D-printed surgical mask that may be disinfected and reused.^8^ In this study, we evaluated practical chemical and thermal decontamination methods for the ability to inactivate SARS-CoV-2, the causative agent of the COVID-19 pandemic, 2 surrogate coronaviruses (MHV and 229E), and a diverse set of clinically relevant human viral pathogens with different viral properties that may influence inactivation.

SARS-CoV-2 and all other viruses tested were completely inactivated by a single application of 10% bleach, ammonium quaternary, and 3% H_2_O_2_ formulations. Also, 70°C dry heat completely inactivated viruses including the SARS-CoV-2 surrogate coronaviruses used (MHV and HCoV-229E); however, this decontamination method was not tested against SARS-CoV-2. Furthermore, 70% IPA and 50°C dry heat did not completely inactivate the viruses included in this study, although viral titers were reduced by >90% (>1 log) with these methods. Notably, in these studies, a single-wipe application of 70% IPA did not decontaminate surfaces completely, and a more stringent application of 70% IPA may be required for complete virus inactivation. To address the concern that blood may alter the efficacy of inactivation, we showed that virus present in 50% whole blood was reduced by >93% when treated with a single application of 10% bleach and ammonium quaternary. These studies illustrate the potential for slight operator differences in inactivation, which highlights this potential importance when suboptimal virus inactivation chemicals (IPA) are utilized.

Ionized disinfecting mist behaves like a gas, and in our studies it was applied to environmental surfaces using 2 types of delivery devices (TOMI Environmental Solutions, trade name SteraMist). This technology is EPA-registered as a hospital disinfectant under the name “Binary Ionization Technology (BIT) Solution,” and appears on EPA lists K, L, G and M. This approach was used because the antimicrobial effect is rapid (15 minutes), there is little damage to materials and surfaces, the H_2_O_2_ dissipates into water vapor and oxygen after application leaving no toxic residue or odor, and the proprietary delivery system is portable, making the adoption of this technology useful.^37^ This approach to disinfection was also completely effective (Fig. 2A–C).

In summary, several decontamination approaches (10% bleach, ammonium quaternary, 3% H_2_O_2_, and 70°C dry heat) were effective on 3D-printed surgical mask materials. Some approaches were effective for inactivation of the SARS-CoV-2 virus, while others were also effective against its surrogates and other clinically relevant viral pathogens. These results are consistent with previous viral inactivation studies, although those studies included variations in decontamination procedures and did not incorporate virus application onto a 3D-printed material.^27,39,40^ The decontamination of 3D-printed surgical masks may be useful during crisis situations in which the supply of surgical masks is limited. Our results may be used to further study decontamination strategies for 3D-printed materials used in clinical settings.
